# CaPSID: A bioinformatics platform for computational pathogen sequence identification in human genomes and transcriptomes

**DOI:** 10.1186/1471-2105-13-206

**Published:** 2012-08-17

**Authors:** Ivan Borozan, Shane Wilson, Paola Blanchette, Philippe Laflamme, Stuart N Watt, Paul M Krzyzanowski, Fabrice Sircoulomb, Robert Rottapel, Philip E Branton, Vincent Ferretti

**Affiliations:** 1 Ontario Institute for Cancer Research, MaRS Centre,South Tower, 101 College Street, Suite 800, Toronto, Ontario M5G 0A3, Canada; 2 Ontario Cancer Institute and the Campbell Family Cancer Research Institute, Toronto Medical Discovery Tower, University of Toronto, 101 College Street, Rm 8-703, Toronto, Ontario M5G 1L7, Canada; 3 Department of Biochemistry, McGill University, McIntyre Medical Building, 3655 Promenade Sir William Osler, Montreal, Quebec H3G 1Y6, Canada; 4 Department of Oncology, McGill University, McIntyre Medical Building, 3655 Promenade Sir William Osler, Montreal, Quebec H3G 1Y6, Canada; 5 The Goodman Cancer Research Centre, McGill University, McIntyre Medical Building, 3655 Promenade Sir William Osler, Montreal, Quebec H3G 1Y6, Canada

## Abstract

**Background:**

It is now well established that nearly 20% of human cancers are caused by infectious agents, and the list of human oncogenic pathogens will grow in the future for a variety of cancer types. Whole tumor transcriptome and genome sequencing by next-generation sequencing technologies presents an unparalleled opportunity for pathogen detection and discovery in human tissues but requires development of new genome-wide bioinformatics tools.

**Results:**

Here we present CaPSID (Computational Pathogen Sequence IDentification), a comprehensive bioinformatics platform for identifying, querying and visualizing both exogenous and endogenous pathogen nucleotide sequences in tumor genomes and transcriptomes. CaPSID includes a scalable, high performance database for data storage and a web application that integrates the genome browser JBrowse. CaPSID also provides useful metrics for sequence analysis of pre-aligned BAM files, such as gene and genome coverage, and is optimized to run efficiently on multiprocessor computers with low memory usage.

**Conclusions:**

To demonstrate the usefulness and efficiency of CaPSID, we carried out a comprehensive analysis of both a simulated dataset and transcriptome samples from ovarian cancer. CaPSID correctly identified all of the human and pathogen sequences in the simulated dataset, while in the ovarian dataset CaPSID’s predictions were successfully validated in vitro.

## Background

Specific viruses have been proved to be etiologic agents of human cancer and cause 15% to 20% of all human tumors worldwide [1]. Moreover, epidemiological studies indicate that new oncogenic pathogens are yet to be discovered [2]. The International Cancer Genome Consortium (ICGC) [3], which intends to study 25 000 tumors belonging to 50 different types of cancer using next generation sequencing technologies, will allow for the first time an in-depth analysis of the viral sequence content of thousands of complete human tumor genomes and transcriptomes. This represents a unique opportunity for the identification of new tumor-associated human pathogens.

However, this opportunity can be fully realized only in conjunction with the development of new genome-wide bioinformatics tools. In this context, several computational approaches have already been developed and successfully applied for the discovery and detection of known and new pathogens in tumor samples [4-9]. We present here CaPSID, a comprehensive open source platform which integrates fast and memory-efficient computational pipeline for pathogen sequence identification and characterization in human genomes and transcriptomes together with a scalable results database and an easy-to-use web-based software application for managing, querying and visualizing results.

## Implementation

CaPSID implements an improved form of a computational approach known as “digital subtraction” [10] that consists of subtracting in silico known human short read sequences from human transcriptome (or genome) samples, leaving candidate non-human sequences to be aligned against known pathogen reference sequences. CaPSID differs from traditional digital subtraction (e.g., [8]), which is used as a filter, eliminating human sequences from the dataset before comparison with pathogen reference sequences. By contrast, CaPSID matches reads against both human and pathogen reference sequences, dividing the reads into three disjoint sets per sample: a set that aligns to pathogen sequences, a set that aligns to both human and pathogen sequences, and a set that does not align to either human or pathogen sequences. This three-way division forms the basis for an exploratory environment for both known and unknown pathogen research.

**Figure 1:**
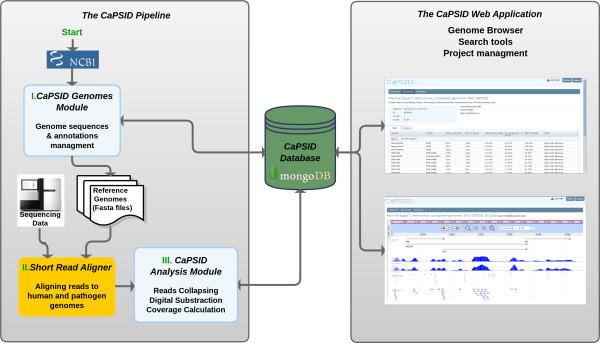
**CaPSID platform.** The CaPSID platform is made of three components: A computational pipeline written in Python for executing digital subtraction, a core MongoDB database for storing reference sequences and alignment results, and a web application in Grails for visualizing and querying the data.

As shown in Figure 1, CaPSID consists of three linked components: 

· A pipeline to analyze and maintain sequencing datasets

· A database which stores reference samples and analysis results

· An interactive interface to browse, search, and explore identified candidate pathogen data

### The CaPSID Pipeline

The CaPSID pipeline is a suite of command-line tools written in Python designed to identify, through digital subtraction, non-human nucleotide sequences in short read datasets generated by deep sequencing of RNA or DNA tumor samples.

The pipeline can be conceptually divided in two distinct modules. The first module, called the Genomes Module, provides users with tools to create and update the in-house reference sequence database required by CaPSID for applying the digital subtraction. It uses BioPython [11] to efficiently parse GenBank files and loads whole genome reference sequences, as well as some of their annotations (e.g. gene and CDS locations), into CaPSID’s database. Our database contains complete sets of human (GRCh37/hg19), viral (4015), microbial (bacterial and archaea) (38035), and fungal (53098) genomes (as of December 2011) from UCSC [12] and NCBI [13]. This module also provides the tools to create customized reference sequence FASTA files needed by short read sequence alignment software.

The second module, called the Analysis Module (see Figure 1), is responsible for executing the digital subtraction and for analyzing its results. It requires two BAM files as input for each sequenced sample to be analyzed: one containing the short read alignment results to the human reference sequences (HRS) and one containing the alignment results to all the pathogen reference sequences (PRS) found in the CaPSID database.

CaPSID can directly process BAM files containing header lines formatted according to NCBI FASTA specification. In order to produce BAM files that can be loaded into CaPSID, a user can also export genome reference files from CaPSID for passing to the short read aligner. Before processing the BAM files, the user can specify a minimum required MAPQ score and all aligned reads that fall below that value will be removed from the analysis. CaPSID can process BAM files containing either single-end or pair-end reads and will automatically determine which analysis method is appropriate. Digital subtraction is not limited to a human reference sequence and can be executed with any host organism as long as its reference sequence has been loaded into the database.

In theory, any short read alignment program - as long as it produces BAM files– is suitable for CaPSID. However, one restriction applies, namely that multiple alignment locations for individual reads must be reported as they can map to more than one pathogen genome. We selected Novoalign (Novocraft Technologies [14]) for its flexibility (allows both mismatches and gaps), accuracy and speed. For ABI SOLiD sequencing data, we chose to use BioScope (Applied Biosystems). Both of these aligners are commercial programs with costly licences. However there are freely available and open source alternatives such as the recently released Bowtie 2 [15] (also allowing both mismatches and gaps) and SHRiMP2 [16].

As output, CaPSID’s digital subtraction implementation produces three disjoint sets of short reads (or records) per sample: a set that aligns to any of the PRS, a set that aligns to both HRS and PRS and a set of reads that does not align to any of the reference sequences (both human and pathogens).

In Algorithm 1 we outline the digital subtraction method used by CaPSID, and how the three disjoint sets are constructed. CaPSID uses the pysam Python module to read and process aligned short read sequences from two input BAM files (*LHRS* and *LNHRS* are read from the same BAM file, in two passes). The algorithm first processes alignment information for each read that maps to the PRS and stores it to the CaPSID’s database (lines 1 to 3). Next, the algorithm processes alignment information for reads that map to HRS, storing it into CaPSID if the same read identifier also maps to the PRS (lines 4 to 8). To avoid memory performance problems, *LHRS*, which may be very large, is processed sequentially. Finally, with another pass through the same BAM file, this time selecting reads that do not align to the human reference sequence, CaPSID identifies unknown reads when they also do not map to the PRS by testing against an in-memory indexed copy of *LPRS*, which is expected to be relatively small (lines 9 to 13).

### Algorithm 1 CapSID’s digital subtraction method

**Require:** input *LPRS* - list of reads that align to the pathogen reference sequence**Require:** input *LHRS* - list of reads that align to the human reference sequence**Require:** input *LNHRS* - list of reads that do not align to the human reference sequence 

1:**for each***item* in *LPRS***do**

2: **add***item***to***PATHOGENSTORE*

3: **end for**

4: **for each***item* in *LHRS***do**

5: **if***item***in***PATHOGENSTORE***then**

6: **add***item***to***HUMANSTORE*

7: **end if**

8: **end for**

9: **for each***item***in***LNHRS***do**

10: **if***item***is not in***LPRS***then**

11: **add***item***to***file(FASTQ)*

12: **end if**

13: **end for**

To better evaluate the significance of the findings, the Analysis Module calculates four different metrics for each sample and for each project as a whole (defined as a collection of samples): *(i)* the total number of aligned reads (or hits) across any given pathogen genome, *(ii)* the total number of hits across genes only within a pathogen genome, *(iii)* the total coverage across each pathogen genome and *(iv)* the maximum coverage across any of the genes in a given pathogen genome. Here we define coverage as the number of genome nucleotides represented in aligned reads normalized by the genome length. Code that calculates the four metrics for each sample has been parallelized to run on multiple processors in order to make these calculations over samples faster.

In addition to the Genome and Analysis Modules, CaPSID includes a command-line script for manipulating FASTQ files that allows the filtering of low quality reads and the removal of duplicates before alignment to reference sequences. The filtering is based on two parameters set by the user, the Phred quality threshold and the number of base pairs allowed below that threshold.

Processing digital subtraction and calculating metrics on two BAM files of 34 GB and 31 GB respectively takes approximately 15 minutes when using 5 GB of RAM on a 16-core AMD 64-bit processor.

### The CaPSID Database

One of the main unique features of CaPSID is that reference sequences and digital subtraction results are both stored as linked data in MongoDB, a scalable, high-performance, open source document-oriented database [17].

The CaPSID database records information on each read identified by the pipeline as part of the two first output sets described above, namely on each read that aligns to either the PRS alone, or to both the HRS and the PRS. The stored fields include, for example, alignment location, length, score, average base qualities, alignment sequence, CIGAR (Compact Idiosyncratic Gapped Alignment Report) information, number of mismatches and whether the read aligns to HRS. Storing both genome and read data makes it possible to run analysis quickly over known gene locations to determine which reads are aligning over gene regions. The third output set of reads identified by the pipeline (reads which do not align to any of the reference sequences) is not stored in the database but is rather saved in FASTQ format to the local file system for further processing.

Next generation sequencing produces large amount of data and because CaPSID stores information about each aligned read and each reference sequence, it needs to deal effectively with large data sizes. CaPSID uses MongoDB [17], a database software that scales horizontally by sharding the database across a cluster of servers, while still enabling fast retrieval of large volume of data. Thanks to this scalable architecture, there is virtually no limit as to how many reads or experiments CaPSID can store in its database. MongoDB reports [17] examples of highly accessed production systems with more than 3.5 TB more than 3.5 TB and over 20 billion records. MongoDB also provides the safety of having no single point of failure, as well as distributing both the processing load and data storage requirements. We tested CaPSID on a single node with more that 25 million read records from 113 different transcriptomes samples without significant drop in its performance. Another advantage of using MongoDB is that it offers API access to a number of programming languages (such as R, Python, Java and more) which, depending on users’ needs, allow a broad range of custom type data analysis.

CaPSID allows also users to store meta-information about each project (defined as a set of samples) and sample. For example, the user can specify the type of disease, the type of cell and sample source together with alignment information (such as the aligner used, sequencing platform, type of sequencing and the location of BAM files) for each sample to be processed.

This database is a key component of the CaPSID platform since it allows users to store, organize and analyze the relevant information from individual samples (derived from the BAM files) in a simple and seamless way, without the burden of manipulating them programmatically.

### The CaPSID Web Application

**Figure 2:**
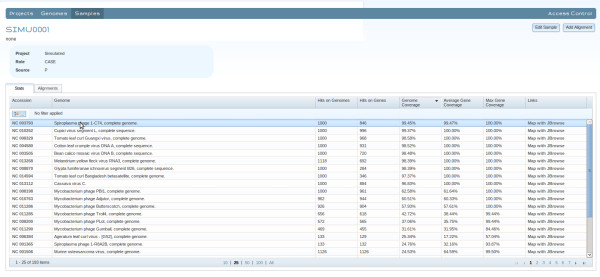
Shows sortable tables of coverage statistics for a sample displayed by CaPSID.

**Figure 3:**
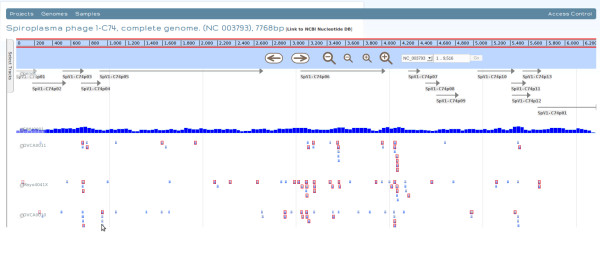
**CaPSID’s integrated genome browser JBrowse.** CaPSID’s integrated genome browser JBrowse, displaying the distribution of read alignments from different samples for a given genome (reads aligning simultaneously to the human reference are shown in red).

After digital subtraction, CaPSID’s web interface enables research attention to be focused on those parts of the sequencing datasets that match the pathogen reference sequences. Its aim is to provide researchers with a rich and interactive interface to manage, query and visualize project results stored in the database. For example, it allows users to search for a specific pathogen and view its statistics across multiple samples or to display sortable tables of coverage statistics for any sample or project (see Figure 2). CaPSID also lets users rank genome hits in a given sample or project by using any of the four metrics described above. CaPSID integrates the genome browser JBrowse [18] to allow, in a simple mouse click, visualizing and analyzing the distribution of read alignments from one or many samples of a given pathogen genome and its genes (see Figure 3). The browser can also differentiate between reads that align PRS only and those that align to both HRS and PRS, allowing users to quickly see which proportion of reads also align to a reference sequence.

CaPSID was designed to be used as a platform for large collaborative projects, such as those between laboratories, and functions as a centralized repository of information. Separating the interface from the analysis pipeline is central to this, as it enables those without direct experience of high-throughput sequencing systems to participate in expert judgements on the digitally subtracted sample datasets.

To enable this, projects in CaPSID are defined as a collection of samples and allow for fine-grained control over user access levels. There are three levels of access to a project: users, collaborators and owners. Users have read-only access to projects, collaborators have permission to add, edit and remove samples and owners have full access to the project, which includes the ability to give permission for others users to access the project, and to remove the project and all associated data entirely from the platform. In addition to specific user level access, projects on the platform can be made public, giving all CaPSID users read-access to the samples.

The CaPSID web application is written in Groovy using the Grails web framework, and allows user authentication and authorization data to be defined in the CaPSID database, an LDAP server or a combination of the two. For example, specific user permissions can be stored in the CaPSID database, while login credentials come from an external LDAP server. This gives full project control to the CaPSID administrators, while still keeping the centralized access control of an LDAP server.

### CaPSID’s manual and documentation

We have provided comprehensive documentation for installing and using CaPSID, complete with a step by step tutorial that takes users from creating a project and loading sequencing data, to analyzing and visualizing aligned reads across pathogen genomes. The full documentation is available at https://github.com/capsid/capsid/wiki.

## Results and discussion

### Analyzing sequencing data using CaPSID

#### Testing CaPSID pipeline accuracy

In order to assess our pipeline and its efficiency to subtract human sequences and detect pathogen ones, we have created a dataset by combining short read sequences from a real human transcriptome sample (publicly available from dbGAP) with sequences simulated at random from 10 viral reference genomes. The publicly available dataset consists of 9 million single-end reads (with read length of 65 base pairs) sequenced from a normal human tissue. We found that 97.3% of reads aligned to the human genome and 0.08% (7241) to viral genomes.

The simulated dataset consisted of 10000 65-mers randomly generated from 10 viral genomes, and 270 reads known to map to both viral and human genomes. The 270 reads were not simulated but selected from one of our own sequenced dataset that has been previously aligned to both the HRS and PRS.

We note that the Novoalign algorithm, before it attempts to align short reads, filters those that have low quality and low complexity by default. For users who want to use other short read aligners that do not include filtering options, CaPSID offers a way of filtering short reads with low quality prior to the alignment (see the CaPSID’s pipeline section). Users should choose their filtering criteria with care as too stringent filtering could eliminate too many reads and produce a drop in sensitivity in detecting viral genomes.

The two datasets (human and simulated) were combined into one single FASTQ file and all short reads were then aligned to the human and viral reference sequences using Novoalign. The two BAM files produced from the alignment step were then processed by CaPSID. CaPSID correctly identified all of the 17511 reads that mapped to viruses (i.e., 7241 from the original human sample and 10270 from the simulated dataset). As explained in the implementation section, CaPSID also keeps a record of reads that align to both human and non-human reference sequences. Our simulated dataset contained 270 of these reads, and CaPSID correctly identified all of them as well.

These results demonstrate the accuracy of our pipeline that is its ability to correctly discriminate all of the alignments results provided by the aligner.

It is worth emphasizing, however, that the alignment accuracy is independent of the CaPSID pipeline and depends entirely on the choice of the alignment algorithm and the quality of the sequenced reads.

The BAM files from this simulated dataset can be downloaded from the CaPSID’s homepage as part of its demo package.

#### Ovarian cancer dataset

Sixteen ovarian tumor transcriptomes were sequenced (for a total of 2.5 billion of reads) at the Ontario Institute for Cancer Research using both the Illumina and AB SOLiD technologies.

Sequenced samples were first aligned to human and pathogen genomes and then processed through CaPSID’s analysis pipeline. After ranking all reported virus genomes in each sample according to the maximum gene coverage metric using CaPSID’s web interface, one sample, OVCA0016, came to our attention.

**Figure 4:**
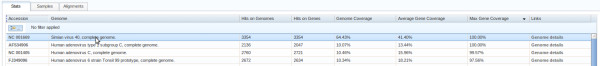
**The top four pathogen genomes hit in the 293T cells as calculated by CaPSID.** The top four genomes hit with the maximum coverage greater than 90% ranked by their maximum gene coverage.

**Figure 5:**
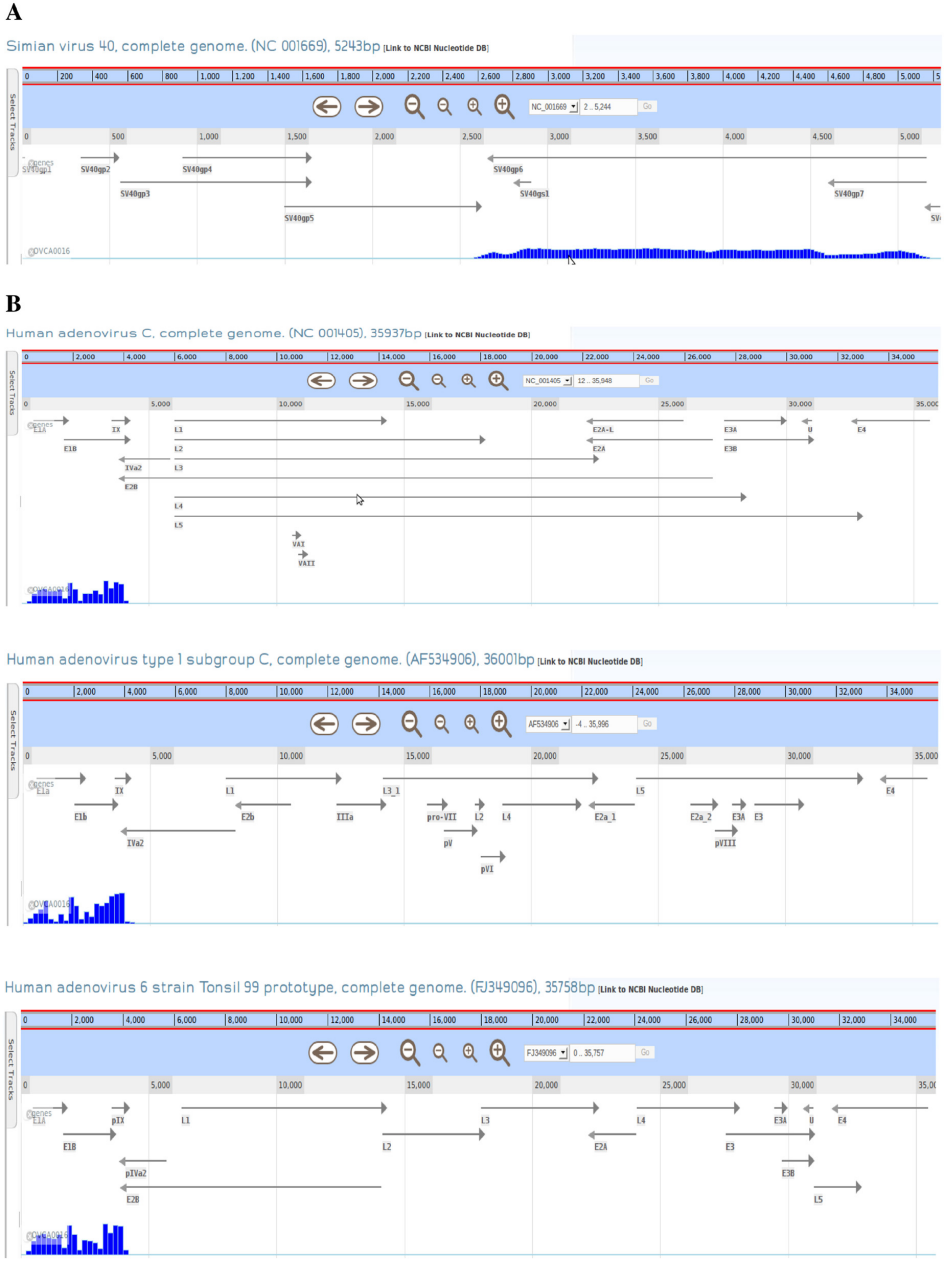
**The distribution of aligned reads across top four pathogen genomes hit in the OVCA0016 cells.A**- Shows the distribution of hits across the SV40 viral genome, with aligned reads concentrating almost entirely across its small and large T-antigens. **B**- Shows E1A and E1B genes to be expressed in all three adenoviruses.

Figure 4 shows that in this sample, among its top four genome hits with gene coverage greater than 90% were the simian virus 40 (SV40) followed by three group C-type human adenovirus genomes. Further analysis using CaPSID’s genome browser revealed that SV40 reads were concentrated almost entirely across its small and large T-antigens (Figure 5A) whereas only the early region 1 E1A and E1B adenovirus (most likely Ad5) genes were expressed (Figure 5B). As it seemed highly unlikely to find a human ovarian tumor expressing both adenovirus and SV40 oncogene products, we hypothesized if the OVCA0016 cell line had been contaminated with or accidentally replaced by 293T cells, an SV40 T antigen-expressing human embryonic kidney cell line [19] derived from Ad5 E1A-/E1B-expressing 293 cells [20].

To process the OVCA0016 sample with approx 255 million reads in two BAM files of 22 GB and 30 GB takes approximately 21 minutes when using 5GB of RAM on a 16-core AMD 64-bit processor.

#### In vitro validation

**Figure 6:**
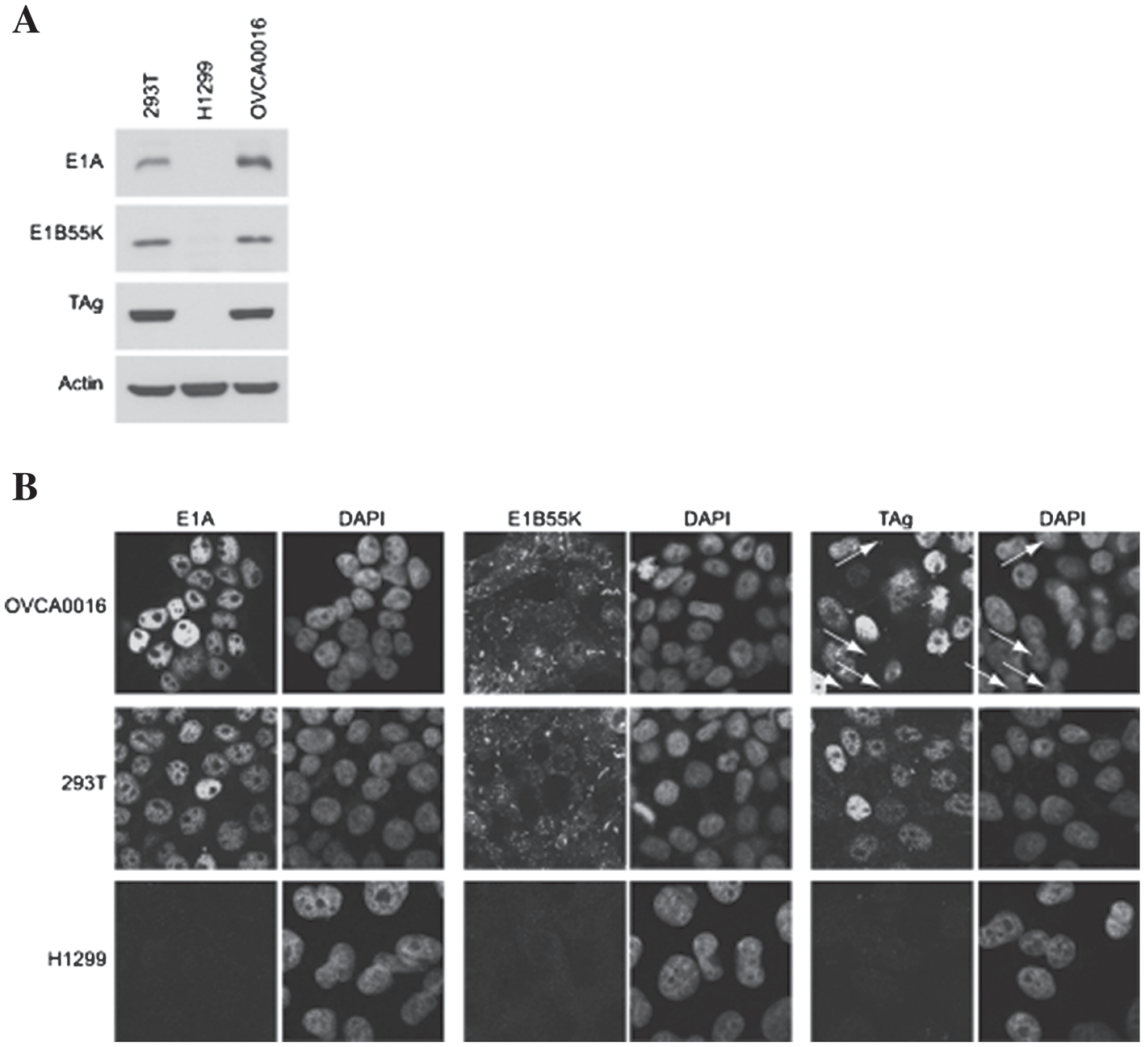
**Analysis of adenovirus E1A/E1B and SV40 T antigen expression in OVCA0016 cultures.A** - Western blotting analysis. Extracts from OVCA0016, 292T and H1299 cells were analyzed by western blotting using M73 (E1A), 2A6 (E1B55K) and Pab101 (SV40 T antigen, BD Pharmingen) antibodies, as described previously [21]. **B** - Immunofluorescence microscopy. The same cells types were grown on coverslips and analyzed by confocal immunofluorescence microscopy [22]. Cells not expressing T antigen have been indicated with arrows on the figure.

To examine this possibility OVCA0016 cells as well as 293T (positive control) and human H1299 lung carcinoma (negative control) cells were harvested in lysis buffer and proteins were separated on SDS-PAGE gels, transferred onto PVDF membranes and probed for Ad5 E1A (Figure 6A top panel), Ad5 E1B55K (middle panel), and SV40 large T (bottom panel), as described previously [21]. With each of these proteins, a signal was detected in OVCA0016 at intensities similar to those found in 293T cells, whereas no signal was observed for either in H1299 cells. The same three cell lines were also grown on cover slips, fixed with 4% paraformaldehyde, stained with the same antibodies and analysed by immunofluorescence on a confocal microscopy as described previously [22]. In each case about 400 cells were analyzed in multiple fields for the expression of E1A protein, E1B55K or SV40 T antigen. Figure 6B shows representative fields examined and that in the case of 293T cells, every cell observed expressed all three viral proteins whereas with H1299 cells none of these species was observed in any cell. Interestingly, with OVCA0016, all cells examined appeared to express both Ad5 E1A and E1B; however, in the case of SV40 T antigen, only one field of all cells examined contained any cells that failed to express this viral protein (marked in the Figure 6B with arrows). As 293T cells are known to multiply in clumps, these results suggested that at most only 1-2% of cells failed to express these viral proteins, suggesting that the OVCA0016 cell line may indeed have been accidentally contaminated and eventually largely overgrown by 293T cells. This hypothesis was then confirmed by interrogation of the DSMZ STR database [23] using 9 short tandem repeat (STR) sequences of OVCA0016 cells generated at the The Centre for Applied Genomics (TCAG). The table provided (see Additional file 1) shows that the top 10 results with the highest arbitrary evaluation value (EV) are 293T and other corresponding variants (HKb20, ProPak-X.36, ProPak-A.52...).

These results indicated that indeed the OVCA0016 cell line appeared to consist almost entirely of 293T cells, with only a small percentage of the original tumor cell line remaining.

Although these results are of no biological significance, this unfortunate contamination of the OVCA0016 ovarian tumor cell line by 293T cells offered an ideal blind test of the efficacy of CaPSID and demonstrated its ability to detect viral gene expression in transcriptome sequences from human tumor cell lines.

#### Assembly of the unaligned reads

As mentioned earlier, CaPSID saves after digital subtraction, the reads that do not align to any of the references sequences in a FASTQ file. The existence of these unaligned reads could be indicative of the presence of some unknown pathogen (or organism) in the sequenced sample. In order to further characterize the unaligned reads in the contaminated sample, we performed de novo assembly of unmapped reads using the short-read assembler Trinity [24].

We note that when assembling whole genome sequencing data (WGS), where the result of alternative splicing is not observed, users should choose a different assembly algorithm such as Velvet [25]. Of the total of 255 million reads, 9 million did not map to any reference sequence in the CaPSID’s database, of these approximately 7.69% assembled into contigs. The Trinity assembler produced in total 13395 contigs ranging from 201 to 6483 bp in length. Using BLAT [26] and MEGA-BLAST [27], we found that only 69 of the assembled contigs did not map to any of the sequences present in either CaPSID’s or NCBI’s reference databases. In order to identify possible novel pathogens, these 69 contigs were screened for the presence of known protein features, using the InterPro [28] database of protein families, domains and functional sites. Nucleic acid sequences were first translated in all 6 frames and then scanned using the InterProScan (iprscan_soappy.py –crc –goterms). We found that none of them displayed a protein feature similar to the pathogen protein features found in the InterPro database. We conclude that none of the remaining contigs are derived from a known pathogen and more likely are due to either sequencing artifacts, uncharacterized regions of the human genome or the presence of some unknown organism having protein features that do not match any of the protein motifs in the InterPro database.

### The comparison of CaPSID to other software for the identification of pathogens in high-throughput sequencing

As mentioned in the background section a number of computational approaches already exist [4-9] for the discovery and detection of known and new pathogens from high-throughput sequencing data. However only two of these, namely PathSeq [6] and RINS [9], are available as integrated open source software similar to CaPSID.

#### Specificity and sensitivity analysis comparison

In this section we compare the accuracy and sensitivity of CaPSID to that of RINS [9], a recently published software for the identification of non-human sequences in high-throughput sequencing datasets.

In order to compare results obtained with CaPSID and RINS we have created a benchmark dataset composed entirely of simulated reads drawn at random from both the human and viral reference sequences. The benchmark dataset is composed of 10 million reads (read length of 100 bp) generated at random from the human reference sequence (GRCh37/hg19) spiked with 10000 reads (read length of 100 bp) generated at random from 10 viral genomes. Each viral genome was then mutated by random substitutions at 3 distinct rates (5%, 10% and 25%). One thousand reads from each mutated genome were then randomly generated to produce an additional 30000 viral reads (1000×10×3). Since the read composition of the benchmark dataset is exactly known it serves as a good standard for evaluating the accuracy and sensitivity of both CaPSID and RINS. In the CaPSID analysis all reads from the benchmark dataset were aligned with the freely available Bowtie 2 [15] aligner. The three metrics used to compare the two software applications are defined in eqs.(1-3) as shown below.

(1) $$\text{Sensitivity}=\frac{n_{\text{vgNOT}}}{n_{\text{vgTot}}}$$

(2) $$\text{Specificity}=\frac{n_{\text{hg}}}{n_{\text{hgTot}}}$$

and the average hit rate ($\bar{\text{hit}\;\text{rate}}$) as

(3) $$\bar{\text{hit}\;\text{rate}}\;(\mathrm{in\%})=\frac{1}{10}\cdot \overset{10}{\underset{g=1}{\sum }}\frac{n_{\text{vgTRU}}E_{g}}{n_{\text{vgTo}}t_{g}}$$

where _*n* *vgNOT*_, _*n* *vgTot*_, _*n* *hg*_, _*n* *hgTot*_, $n_{\mathrm{vgTRU}E_{g}}$ and $n_{\text{vgTo}t_{g}}$ are defined as shown below 

· nvgNOT = # of reads derived from viral genomes mapping to the human reference

· nvgTot = total # of reads derived from viral genomes

· nhg = # of reads derived from the human genome mapping to the human reference

· nhgTot = total # of reads derived from the human genome

· $n_{\mathrm{vgTRU}E_{g}}$ = # of reads derived from the viral genome g mapping to the viral reference sequence g

·$n_{\mathrm{vgTo}t_{g}}$ = total # of reads derived from the viral genome g

We found that the sensitivity and specificity of both CaPSID and RINS were 100% on the benchmark dataset (including both the non-mutated and mutated data). The results of our analysis indicate that CaPSID gives very similar results to those of RINS in terms of sensitivity, specificity and performance (CaPSID runs in < 20 min and RINS in < 14 min when performing the analysis on the benchmark dataset containing ≈ 10 million reads on a 16-core AMD 64-bit processor using < 6GB or RAM). However when using our third metric (see eq.3) we find that CaPSID performs better than RINS by mapping significantly more reads derived from mutated genomes back to their original reference sequences as shown in Table 1. This result demonstrates the ability of our approach to identify viral genomes with substantially divergent sequences (up to 25%) and indicates that it could be used for the identification of novel pathogen with up to 25% homology to the reference sequences stored in its database.

**Table 1:** Comparison of the average hit rate between CaPSID and RINS for the three distinct mutation rates

	**0%**	**5%**	**10%**	**25%**
$\bar{\mathrm{hit}\;\mathrm{rate}}$**(CaPSID)**	100%	95.51%	65.39%	1.11%
$\bar{\mathrm{hit}\;\mathrm{rate}}$**(RINS)**	100%	66.21%	19.60%	0.20%

#### Other feature comparison

In addition to the comparison in accuracy and performance presented above, CaPSID includes additional important features that to the best of our knowledge are not part of either PathSeq or RINS. 

· Unlike PathSeq or RINS, which are primarily analysis tools, CaPSID has an easy to use and manageable database allowing users to organize, store, analyze and visualize information from each project and sample in a seamless way. CaPSID’s database is accessible by CaPSID’s user-friendly web application that integrates a genome browser (complete with genome annotations) making the analysis and visualization of alignment results straightforward.

· CaPSID provides coverage metrics that allow users to rank pathogens in significance based not only the overall genome coverage but on the gene coverage as well.

· In CaPSID, users can align short reads using any aligner of their choice: having a single preferred aligner might be a limiting factor especially as new faster and more accurate aligners become available (for example PathSeq uses the MAQ aligner followed by Mega BLAST and BLASTN in order to align those reads with additional mismatches or gaps that are not aligned by MAQ (see [6]) and RINS [9] uses a combination of BLAT and Bowtie).

· Unlike PathSeq or RINS, CaPSID does not wholly remove reads that simultaneously map to both human and pathogen genomes but keeps them in the database allowing rapid identification of pathogen-to-host integration sites.

· CaPSID does not require a third party commercial computing platform such as the one used by PathSeq (Amazon Elastic Compute Cloud, EC2) and can be run efficiently on either a desktop computer or a cluster.

PathSeq performs subtractive alignments using six different human genomes and then uses local aligners such as Mega BLAST and BLASTN to re-align reads (not aligned by MAQ during the initial alignments) to the two additional human sequence databases. In comparison, CaPSID’s current subtraction is done in one pass against a single human reference genome with splice junctions. Thus CaPSID might potentially fail to subtract some of the reads aligned by PathSeq to its database of human references– although this is not a fundamental constraint of the CaPSID architecture, and further work to use multiple human reference databases would be relatively straightforward. However, because PathSeq uses local aligners its approach can be computationally very intensive and for users desiring to identify known pathogens with a large reduction in runtime CaPSID’s approach might be more advantageous, especially when handling the large datasets of next generation sequencing. In addition, CaPSID’s three-way approach of retaining reads that map to pathogen genomes, even when they also map to human genomes, reduces the risk that pathogen reads are inadvertently omitted during subtraction by both PathSeq or RINS.

## Conclusions

In this article, we have presented CaPSID– a comprehensive bioinformatics platform for the detection of pathogen sequences in genome and transcriptome samples. We have demonstrated that CaPSID is an efficient tool that performs well on both the simulated and real datasets. We have shown that CaPSID’s predictions can be successfully validated in vitro, and that CaPSID offers new and useful features that are not available in any current software used for the identification of pathogens in high-throughput sequencing. Furthermore and more importantly, CaPSID is suitable for collaborative types of projects between teams of scientists, for example between bioinformaticians and molecular virologists, through its web interface allowing researchers without expert knowledge in computational techniques to analyze alignment results stored in the CaPSID’s database. The CaPSID platform is currently used in real production environment to analyze sequencing data generated by the OICR laboratories from different tumor types. Since CaPSID was deployed at the OICR, it was used in the analysis of more than 113 transcriptome samples across six different projects with a total of 25 million aligned reads stored in the CaPSID database. We believe CaPSID to be a versatile research tool that we hope will be used by researchers for the detection and identification of known and new pathogens using next generation sequencing data.

## Availability and requirements

**Project Name:** CaPSID **Project home page:**https://github.com/capsid/capsid**Operating system(s):** Linux, Mac OS X **Programming language:** Python 2.7 (no support for Python 3 at the moment), Groovy**Other requirements:** MongoDB, OpenJDK (≥ 1.6.0_20), BioPython **License:** GNU GPL3 **Any restrictions to use by non-academics:** None

## Competing interests

The authors declare that they have no competing interests.

## Authors’ contributions

VF, IB, SW and PL developed CaPSID, VF and IB set up the methodology, IB carried out data analysis and drafted the manuscript, SW implemented CaPSID. VF drafted the manuscript, conceived and directed the project. PEB, PB and RR designed and performed in vitro validation experiments. PMK and FS carried out the STR analysis. VF, IB, SW, PMK, FS, SNW, PB and PEB wrote and revised the manuscript. All authors read and approved the final manuscript.

## Supplementary Material

Additional file 1 Table S1. Short tandem repeat sequence (STR) analysis in OVCA0016. Table S1 shows that the top 10 results with the highest arbitrary evaluation value (EV) are 293T and other corresponding variants (HKb20, ProPak-X.36, ProPak-A.52...). Click here for file
